# Design of a Distance-Compensated Infrared Thermometer for Canopy Temperature Monitoring

**DOI:** 10.3390/s26144380

**Published:** 2026-07-10

**Authors:** Liem H. T. Nguyen, Manu Chilukuri, Christopher D. Delhom, Prasanna Gowda, Sungyong Jung, Hyusim Park

**Affiliations:** 1 McComish Department of Electrical Engineering and Computer Science, South Dakota State University, Brookings, SD 57007, USA; liem.nguyen@jacks.sdstate.edu (L.H.T.N.); sungyong.jung@sdstate.edu (S.J.); 2 Department of Electrical Engineering, University of Texas at Arlington, Arlington, TX 76019, USA; 3 National Center for Alluvial Aquifer Research, USDA Agricultural Research Service, Stoneville, MS 38776, USA; chris.delhom@usda.gov; 4 Smart Agriculture Research Center, University of Texas at Arlington, Arlington, TX 76019, USA; prasanna.gowda@uta.edu; 5 Department of Electrical Engineering, University of North Texas, Denton, TX 76203, USA

**Keywords:** surface temperature, distance compensation, infrared thermometry, precision agriculture, ultrasonic sensor

## Abstract

Infrared (IR) thermometry is essential for non-contact surface temperature monitoring in precision agriculture, but conventional single-pixel devices exhibit distance-dependent measurement errors that hinder field deployment. Most existing solutions require either fixed-geometry installations, post-processing corrections, or expensive thermal imaging systems with computational overhead. This paper presents the design, development, and evaluation of an IR thermometer measurement system that implements a lightweight signal processing algorithm for distance compensation. The instrumentation module integrates the thermometer’s temperature signal with ultrasonic distance measurements and employs a distance-bias lookup table (LUT) calibrated at regulated setpoints. Real-time signal conditioning through bilinear interpolation generates corrected temperature outputs. Under controlled reference-target conditions, system evaluation across 5–200 cm shows that uncompensated errors reach approximately −5 °C at 200 cm, whereas the proposed signal processing reduces the distance-dependent residual RMSE to below 0.20 °C over the tested range. The instrument also preserves short-term repeatability, with standard deviations between 0.08 °C and 0.12 °C across stand-off distances from 20 to 200 cm. By delivering distance-invariant measurements through external signal processing, the system is designed to support flexible, height-independent deployment of single-pixel IR thermometry for precision agriculture applications such as canopy temperature monitoring. A preliminary field measurement on a live tree canopy under one overcast condition provides initial evidence that the distance-dependent drift can be reduced outdoors, with the compensated output remaining within 0.7 °C of an external handheld reference across the 5–200 cm range; comprehensive multi-condition field validation remains necessary in future work.

## 1. Introduction

Precision agriculture increasingly relies on continuous monitoring of plant physiological status to optimize resource allocation and maximize yields. Infrared (IR) thermometry stands out as a particularly valuable sensing method, offering noninvasive assessment of plant conditions including plant stress and evapotranspiration [1]. However, these sensors face a significant challenge: their apparent temperature reading can exhibit distance-dependent bias. As the field of view (FOV) expands at greater distances, the sensor inevitably captures mixed radiation from both the target canopy and the surrounding background [2,3,4]. This limitation is especially critical in agricultural applications, where canopy temperature monitoring reveals water stress [5,6,7], disease onset [8,9], and heat damage. Distance-induced errors can reach several degrees Celsius [10,11], severely restricting deployment flexibility and compromising measurement reliability.

The physics behind these distance errors stems primarily from FOV expansion and the resulting changes in the target-to-background ratio within the measurement footprint. Yet existing solutions inadequately address this challenge for real-time field deployments. Thermal imaging systems can bypass the issue through high spatial resolution, but their costs remain prohibitive for routine monitoring [12,13,14]. Fixed-geometry installations avoid distance variation entirely, yet sacrifice the flexibility needed for diverse crop architectures and growth stages [15,16]. Post-processing correction methods cannot provide the real-time capability that autonomous monitoring systems require for immediate control decisions [17,18]. To the authors’ knowledge, real-time, distance-invariant measurement through integrated signal processing remains uncommon among single-pixel IR thermometer implementations, a gap that restricts broader deployment of this otherwise mature sensing technology; Section 5 contrasts the proposed approach with representative recent systems.

Recent advances in agricultural instrumentation have explored various strategies to enhance IR thermometry accuracy. Arduino-based thermopile arrays can achieve spatial averaging comparable to expensive thermal cameras, though they require complex data fusion algorithms unsuitable for resource-constrained embedded systems [19,20]. Center pivot-mounted systems combine GPS positioning with IR sensors and ultrasonic rangefinders to generate georeferenced temperature maps, demonstrating multi-sensor integration but relying on existing irrigation infrastructure [21]. Hybrid RGB-thermal systems use sophisticated image segmentation for background compensation, yet add computational overhead that prevents continuous real-time operation [22,23,24]. Indoor monitoring solutions successfully integrate low-cost thermopile arrays into IoT networks, although such controlled environments naturally minimize the distance variation challenges encountered in open-field conditions [25].

Beyond these, mobile phenotyping platforms have begun incorporating ultrasonic rangefinders for geometric modeling [26,27]. These systems validate distance measurements’ potential for improving IR thermometry but do not implement real-time signal conditioning for distance compensation. This pattern reveals a critical gap across all current approaches: the absence of integrated signal processing for geometric correction [28,29]. Whether through sensor arrays, computational segmentation, or multi-sensor fusion, existing systems treat distance-induced bias as a constraint to avoid rather than a systematic error to correct [30,31]. Without real-time compensation algorithms, single-pixel IR thermometers, despite their advantages in cost, power consumption, and simplicity, can exhibit substantial distance-dependent bias under varying geometric conditions. The underlying sensors possess adequate precision and stability; they require only appropriate signal processing to eliminate distance-dependent systematic bias [32].

To address this measurement challenge, this paper presents a distance-compensated IR thermometer system that achieves real-time correction through integrated signal processing. The proposed approach combines infrared temperature sensing with ultrasonic distance measurement, employing a calibrated lookup table with bilinear interpolation for continuous correction. The signal processing algorithm, developed through systematic characterization at regulated temperature setpoints, transforms distance-dependent measurements into distance-invariant outputs across typical agricultural monitoring ranges. The instrumentation module operates autonomously, logging temperature, distance, and location data with synchronized timestamps. Users mount the system above the measurement target without stringent positioning requirements, as the distance compensation maintains a distance-invariant reading throughout the 5–200 cm operating range. By implementing compensation entirely through lightweight embedded processing, the system achieves the distance-invariance of fixed-geometry installations while retaining deployment flexibility.

## 2. System Design

**Figure 1:**
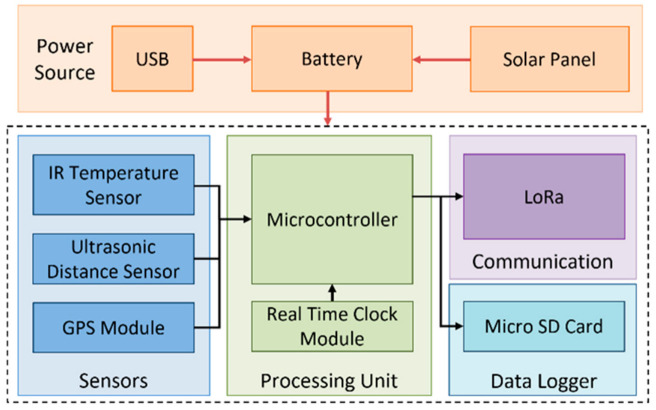
Proposed system block diagram.

The distance-compensated IR thermometer system employs a modular architecture comprising four functional blocks such as sensors, a processing unit, power management, and a communication interface. Figure 1 presents the system block diagram illustrating the interconnections between these subsystems.

The sensor suite integrates three primary measurement components. The single-pixel IR thermometer, MLX90614KSF [33], provides non-contact temperature measurements, featuring a 35° field of view and a manufacturer specified accuracy of ±0.5 °C and a measurement resolution of 0.02 °C in the −40 °C to 125 °C object temperature range. Standoff distance is measured by a US-100 ultrasonic rangefinder [34] (2.4–5.5 V supply, <15° beam angle, 2–450 cm range, ±(0.3 cm + 1%) accuracy) operating in temperature-compensated UART mode, in which an on-board temperature sensor corrects the speed-of-sound term of each distance reading, thereby mitigating ambient-temperature sensitivity of the ranging measurement. The u-blox NEO-M9N GPS module [35] provides latitude and longitude coordinates for georeferencing temperature measurements in agricultural mapping applications.

The processing unit utilizes an ATSAMD21G18A [36] ARM Cortex-M0+ microcontroller operating at 48 MHz, managing sensor acquisition, compensation calculations, and system coordination. A DS3231 [37] real-time clock IC maintains accurate timestamps with battery backup, ensuring temporal data integrity during power cycles. The microcontroller interfaces with an OLED display and three-button interface for field configuration, allowing users to adjust measurement intervals and monitor system status without external devices.

The power subsystem accepts either USB or solar input to charge the battery and supplies a regulated 3.3 V to all components. Controllable power lines on the GPS receiver, the LoRa transceiver, and the sensors allow the controller to duty cycle peripherals. The firmware supports configurable sampling intervals to balance measurement frequency against power consumption.

In continuous logging mode, the IR sensor remains powered to preserve thermal equilibrium (2.5 mA), while the GPS receiver, LoRa transceiver, and OLED display are switched off through their controllable supply lines between measurements. With the SD card in standby (0.2 mA) and the microcontroller in low-power sleep between samples, the baseline current is approximately 2.7 mA, and each logged measurement requires roughly 1 s of active acquisition at about 24 mA. On a 2500 mAh battery, the resulting projected operating lifetime is approximately 38 days at the default 15-min sampling interval, decreasing to about 34 days at the 1-min minimum interval, since the always-on infrared sensor and SD-card standby dominate the energy budget.

Communication and data storage employ dual pathways for reliability. An RFM95W [38] LoRa transceiver operating at 915 MHz enables wireless data transmission to remote gateways, while a microSD card provides local storage in CSV format. All data is continuously written to local storage while simultaneously being transmitted wirelessly. If connectivity is unavailable, the system queues the unsent data for later transmission, ensuring a complete and permanent local record is always maintained.

**Figure 2:**
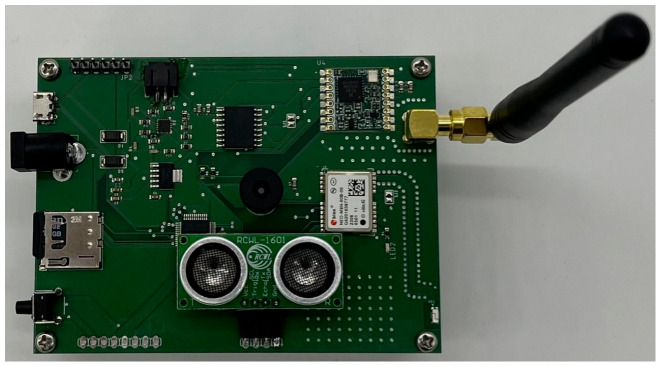
Printed circuit board implementation (9 × 7 cm) showing component layout.

Figure 2 shows the printed circuit board implementation measuring 9 × 7 × 2 cm. The design positions the ultrasonic transducers and IR sensor aperture on the same edge for aligned distance and temperature measurements. The GPS and LoRa antenna connectors are separated to minimize RF interference.

## 3. Firmware Implementation

The firmware implements a real-time signal conditioning pipeline that converts the raw readings into distance-invariant temperatures. At each sampling interval, a real-time clock interrupt wakes the microcontroller. Five successive ultrasonic distance readings are acquired, and a median filter is applied to obtain a stable stand-off distance d (cm). The IR sensor is read to obtain the raw temperature $T_{raw}$ (°C). Calibration is organized as a two-dimensional lookup table (LUT) of distance-dependent bias over a uniform distance grid and a set of regulated temperature setpoints. Let the distance grid be $d_{m}$ for *m* = 0, …, 39, with $d_{m}$ ranging from 5 to 200 cm at 5 cm intervals. The temperature anchors are $T_{k}$ for *k* = 1, …, 10, with $T_{k}$ ranging from 10 °C to 100 °C in 10 °C increments. For each calibration pair ($d_{m},T_{k}$), the tabulated value is

(1) $$e\left[k,m\right]=T_{raw}\left(d_{m},T_{k}\right)-T_{k}$$

At run time, the correction for the new measurement (d, $T_{raw}$) is obtained by piecewise linear interpolation in distance and temperature. First, find *m* and *k* such that $d_{m}\leq d\leq d_{m+1}$ and $T_{k}\leq T_{raw}\leq T_{k+1}$ to compute the distance interpolation weight:

(2) $$\alpha_{d}=\frac{d-d_{m}}{d_{m+1}-d_{m}}$$

The distance-interpolated errors at the bounding temperature indices are

(3) $${\tilde{e}}_{k}\left(d\right)=\left(1-\alpha_{d}\right)\times e\left[k,m\right]+\alpha_{d}\times e[k,m+1]$$

Next, compute the temperature interpolation weight:

(4) $$\alpha_{T}=\frac{T_{raw}-T_{k}}{T_{k+1}-T_{k}}$$

and blend the distance-interpolated errors to obtain the final correction:

(5) $$e\left(d,T_{raw}\right)=\left(1-\alpha_{T}\right)\times {\tilde{e}}_{k}\left(d\right)+\alpha_{T}\times {\tilde{e}}_{k+1}\left(d\right)$$

The compensated temperature is then calculated as in

(6) $$T_{comp}=T_{raw}-e(d,T_{raw})$$

Inputs falling outside the calibrated intervals (5–200 cm in distance; 10–100 °C in temperature) are clamped to the nearest boundary before interpolation. With the uniform grid, both interpolations reduce to two linear blends per sample, keeping the per-sample computational cost small. The measurement window consists of peripheral power-up and stabilization, acquisition of IR and ultrasonic readings, digital filtering of the distance estimate, linear interpolation, and final compensated temperature computation. The result is written to the microSD card in CSV format. When LoRa wireless communication is available, the result is also transmitted to the nearest gateway. At deployment, a startup warm-up of 120 s precedes logging so that the continuously powered IR sensor reaches thermal equilibrium; cold-start characterization showed that the compensated reading settles to within ±0.20 °C within 104 s at the most demanding 60 °C setpoint, after which only steady-state measurement noise remains. Because the IR sensor is not power-cycled during operation, no per-reading warm-up is required.

Although the LUT is calibrated from 10 °C to 100 °C to stabilize interpolation and facilitate future extensions, the performance metrics reported in this paper are validated at 15 °C, 30 °C, and 60 °C only, corresponding to the agricultural operating band of interest. Readings outside the validated band are excluded. The methodology, however, is readily extendable by incorporating additional calibration setpoints and regenerating the LUT.

## 4. System Evaluation Method

To verify and evaluate the proposed system, controlled experiments were conducted at three reference temperatures, 15 °C, 30 °C, and 60 °C, chosen to span the agricultural operating band of interest. The 15 °C and 30 °C setpoints represent baseline-to-optimal canopy conditions: mean growing temperatures near 15 °C have been reported as a non-stressed benchmark for grain yield [39,40], while optimal soil and canopy temperatures for warm-season crops fall in the 23–30 °C range [41,42]. Resolving sub-degree differences with low variance at these setpoints is therefore relevant to detecting incipient water stress or disease.

The 60 °C setpoint probes the upper extreme. Although foliar damage thresholds are typically lower, exposed soil and surface temperatures in agricultural settings have been reported to exceed 50 °C and approach 70 °C [43,44,45,46,47,48], so 60 °C represents a demanding high-temperature condition for characterizing the instrument rather than a typical canopy value. Because the system was evaluated on a reference target rather than on plants, these setpoints are used to bound instrument performance over the relevant temperature span, not to reproduce specific crop physiology; as noted in Section 3, the firmware LUT spans 10–100 °C while performance is validated at these three setpoints.

### 4.1. Distance Compensation Experiments

**Figure 3:**
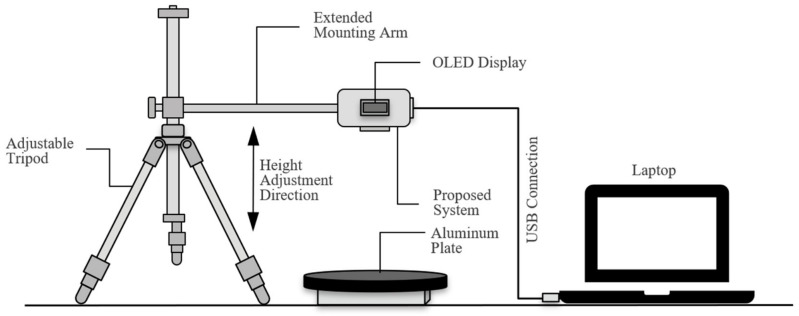
Bench configuration for the distance-compensation experiments. The proposed system is mounted on an adjustable tripod via an extended arm above the regulated matte-black aluminum reference plate; the stand-off distance is set by the tripod column height, and data are logged to a laptop over USB.

Distance sweep tests were performed with a thermally regulated, high emissivity matte black aluminum plate as the reference target. All calibration and evaluation experiments were conducted indoors at an ambient air temperature of approximately 23 °C and approximately 40% relative humidity, in a windowless laboratory with no direct sunlight on the instrument or the target. Only the target plate temperature was regulated, and the plate temperature was verified with a Fluke 59 MAX infrared thermometer (Fluke, Everett, WA, USA) (nominal accuracy ±2.0 °C or ±2.0% of reading). The matte black plate provides a spatially uniform, high-emissivity reference that isolates the distance-dependent bias under controlled conditions; it does not reproduce the radiometric heterogeneity of a plant canopy (variable leaf angles, canopy gaps, soil background, emissivity variation, and wind-driven motion), so the compensation characterized here applies to a uniform target of known geometry, and extension to heterogeneous canopies will require dedicated field characterization. The plate, with a radius of 30 cm, was mounted normal to the instrument’s optical axis. Thermal regulation was achieved using a hot plate for the 30 °C and 60 °C setpoints, while a Peltier module with a temperature controller was used to set the plate at 15 °C for data collection. The instrument comprised the single-pixel IR thermometer and a co-aligned ultrasonic range sensor for stand-off measurement. The assembly was mounted on an adjustable tripod whose column height set the stand-off distance, at nominal 5 cm intervals from 5 to 200 cm, verified with a tape measure (Figure 3). At each distance (where i indexes the 40 measurement positions), and each reference temperature (15 °C, 30 °C, 60 °C), the IR raw temperature $T_{raw}$, the compensated temperature $T_{comp}$, and the reference temperature $T_{ref}$ were logged. For analysis, uncompensated and compensated errors were computed as

(7) $$\varepsilon_{raw}\left(d_{i}\right)=T_{raw}\left(d_{i}\right)-T_{ref}$$

(8) $$\varepsilon_{comp}\left(d_{i}\right)=T_{comp}\left(d_{i}\right)-T_{ref}$$

To quantify performance across the operating points, distances were partitioned into five contiguous ranges of 0–40, 40–80, 80–120, 120–160 and 160–200 cm. For each range $R_{j}$, the root-mean-square error (RMSE) of the compensated and the uncompensated measurements were computed as

(9) $${RMSE}_{comp}(R_{j})=\sqrt{\frac{1}{N_{j}}\sum {\varepsilon_{comp}\left(d_{i}\right)}^{2}}$$

(10) $${RMSE}_{uncomp}(R_{j})=\sqrt{\frac{1}{N_{j}}\sum {\varepsilon_{raw}\left(d_{i}\right)}^{2}}$$

where $N_{j}$ is the number of samples within $R_{j}$.

### 4.2. Repeatability Experiments

Precision and stability were assessed at three stand-off distances of 20, 100, and 200 cm with the system rigidly mounted normal to the reference target. At each reference temperature of 15 °C, 30 °C and 60 °C, *n* = 300 consecutive compensated measurements were acquired at a rate of 1 sample per second. For each run, the error sequence, $\varepsilon_{i},$ the sample mean error, $\bar{\varepsilon }$, and the sample standard deviation, SD, were computed as in (11), (12), and (13) respectively.

(11) $$\varepsilon_{i}=T_{comp}\left(t_{i}\right)-T_{ref}$$

(12) $$\bar{\varepsilon }=\frac{1}{n}\sum_{i=1}^{n}\varepsilon_{i}$$

(13) $$SD=\sqrt{\frac{1}{n-1}\sum_{i=1}^{n}{\left(\varepsilon_{i}-\bar{\varepsilon }\right)}^{2}}$$

Error histograms with 0.05 °C bin width were constructed to visualize the noise distribution and assess approximate normality.

## 5. Results and Discussion

**Figure 4:**
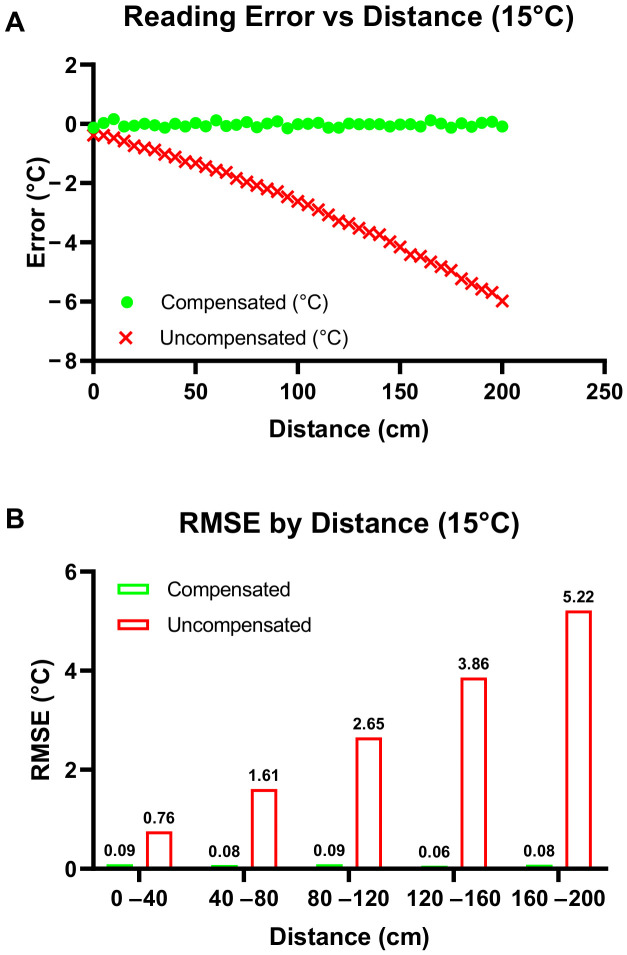
Distance-dependent error at 15 °C: (**A**) error vs. distance (raw and compensated); (**B**) RMSE by distance.

Figure 4 summarizes the distance-dependent error characteristics at the 15 °C reference temperature and demonstrates the effectiveness of the compensation. In Figure 4A, uncompensated measurements exhibit a pronounced negative bias that increases approximately linearly with distance, starting near zero at close range and reaching about −6.0 °C at 200 cm. This trend indicates a systematic distance-dependent bias in the uncompensated single-pixel IR measurement. In contrast, the compensated measurements remain tightly clustered around zero error across the full 0–200 cm sweep, with deviations confined to within approximately ±0.2 °C regardless of distance. Figure 4B quantifies these effects via RMSE computed over five distance bins. Uncompensated RMSE rises monotonically from 0.76 °C to 1.61 °C, 2.65 °C, 3.86 °C, and 5.22 °C, respectively. The compensated RMSE values are 0.09 °C, 0.08 °C, 0.09 °C, 0.06 °C, and 0.08 °C, yielding significant improvement factors from near to far range.

**Figure 5:**
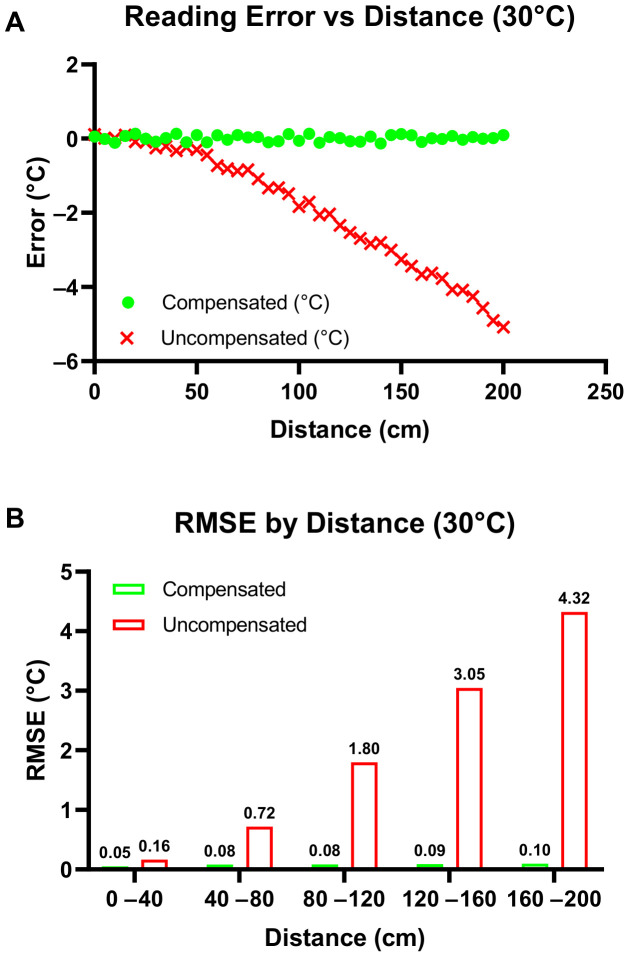
Distance-dependent error at 30 °C: (**A**) error vs. distance (raw and compensated); (**B**) RMSE by distance.

Figure 5 presents the distance-dependent error characteristics at the 30 °C setpoint. In Figure 5A, uncompensated measurements exhibit a pronounced negative bias that increases approximately linearly with distance, reaching about −5.2 °C at 200 cm. A similar systematic distance-dependent bias is observed at this setpoint. In contrast, the compensated measurements remain tightly clustered around zero error across the full 5–200 cm sweep, with deviations confined to within ±0.2 °C regardless of distance. Figure 5B quantifies these effects via RMSE computed over five distance bins. Uncompensated RMSE rises monotonically from 0.16 °C to 0.72 °C, 1.80 °C, 3.05 °C, and 4.32 °C, respectively. The compensated RMSE values are 0.05 °C, 0.08 °C, 0.08 °C, 0.09 °C, and 0.10 °C, yielding improvement factors from near to far range.

**Figure 6:**
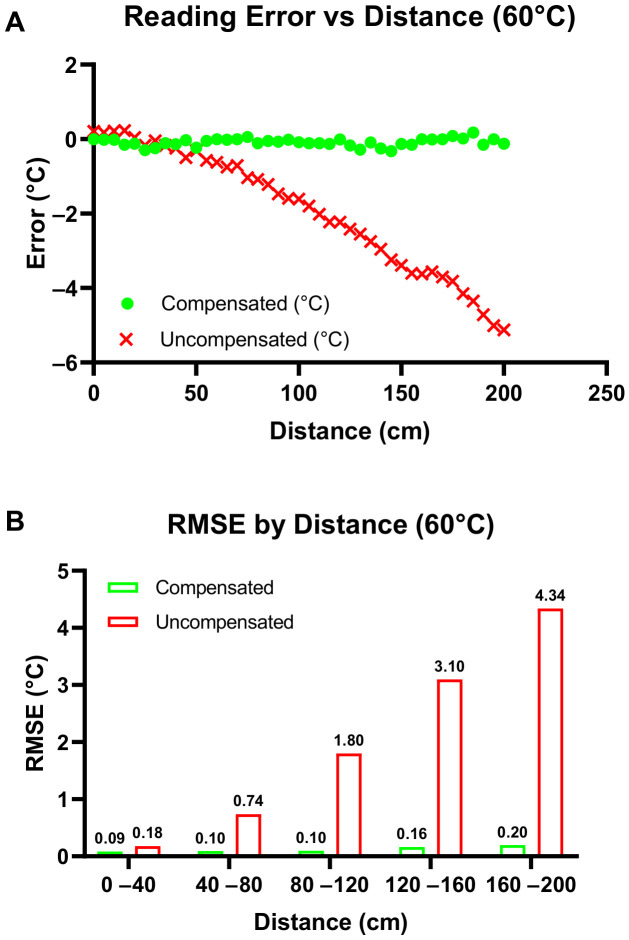
Distance-dependent error at 60 °C: (**A**) error vs. distance (raw and compensated); (**B**) RMSE by distance.

Figure 6 reports the corresponding analysis at 60 °C. The uncompensated error in Figure 6A follows a similar distance trend with negative distance bias reaching −5.1 °C at 200 cm, indicating that the dominant bias arises from target to background mixing rather than temperature-specific effects. After compensation, the error remains bounded within ±0.3 °C across the 5–200 cm range, with slightly increased scatter compared to 30 °C. The RMSE results in Figure 6B mirror this behavior with uncompensated RMSE increases from 0.18 °C to 0.74 °C, 1.80 °C, 3.10 °C, and 4.34 °C across the five distance bins, whereas compensated RMSE values are 0.09 °C, 0.10 °C, 0.10 °C, 0.16 °C, and 0.20 °C. The modest growth in the compensated RMSE at 60 °C is consistent with larger target-to-background temperature contrasts and radiance nonlinearity, but the residual RMSE remains below 0.20 °C across the evaluated distance range.

Across the three setpoints, the uncompensated distance error appears to result from a combination of field-of-view underfill, the angular response of the single-pixel IR sensor, and radiance from the surrounding background. As the sensor is moved farther from the target, the target occupies a smaller portion of the sensor footprint, and the measured temperature becomes more dependent on the specific target geometry and background condition. For this reason, the correction is specific to the calibrated target configuration and would need to be recharacterized for targets with different geometry or background contrast.

To confirm that these results reflect genuine compensation performance rather than reproduction of the calibration points, the distance compensation was assessed by leave-one-out cross-validation. Each interior grid distance was removed one at a time from the lookup table and reconstructed by interpolation from the two neighboring grid points. Boundary distances were handled using one-sided interpolation from the nearest available grid points. Across all three setpoints, the held-out compensated RMSE was 0.081 °C with a worst-case residual of 0.235 °C, comparable to the in-grid values reported above. Because calibration and evaluation therefore rely on independent points, the reported sub-0.20 °C RMSE is not an artifact of evaluating on the calibration grid; this analysis additionally bounds the lookup-table interpolation error at non-grid distances.

**Figure 7:**
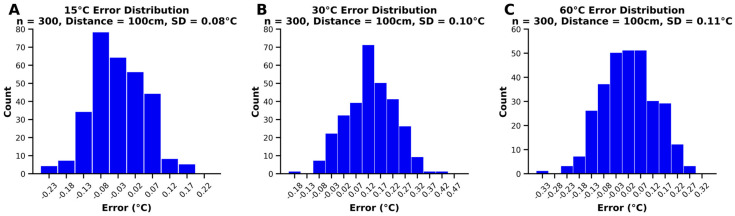
Error distributions at 100 cm: (**A**) at 15 °C; (**B**) at 30 °C; (**C**) at 60 °C.

Figure 7 presents the compensated-error distributions at 100 cm for the three reference setpoints. The distributions are approximately Gaussian and centered near zero, with standard deviations of 0.08 °C at 15 °C, 0.10 °C at 30 °C, and 0.11 °C at 60 °C. To verify that short-term stability was maintained across the operating distance range, the repeatability test was also performed at representative short- and long-range stand-off distances of 20 and 200 cm. As summarized in Table 1, the compensated output remained stable across all evaluated setpoints and distances, with standard deviations between 0.08 °C and 0.12 °C. The modest increase in variation at longer distances and higher target temperature is consistent with increased sensitivity to underfilled-field-of-view effects, angular optical response, and target/background radiance mixing. These results confirm stable short-term behavior at a 1 Hz sampling rate across the 20–200 cm range. These controlled-target repeatability results show that the instrument has stable short-term output under the tested laboratory conditions. However, they should be interpreted as repeatability of the compensated measurement, not as validated canopy-temperature measurement accuracy under heterogeneous field conditions.

The growth of compensated RMSE at 60 °C, and its mild increase with distance, follows from the physics of the underfilled field of view. Emitted radiance rises steeply with absolute temperature, so a fixed fractional underfill of the 35° field of view (about 17° of subtense for the 60 cm target at 200 cm) produces a larger absolute radiance discrepancy when the target is hot relative to its background. The lookup table removes the mean distance-dependent bias, but the residual scatter scales with the target-to-background contrast, which is largest at 60 °C and at long range. This also delimits the method: the correction is calibrated for a target of known geometry against a stable background, so a strongly contrasting or non-uniform background would shift the bias surface and require recharacterization. It is therefore the systematic distance bias, not the sensor’s intrinsic precision, that the compensation addresses. The manufacturer’s ±0.5 °C figure is an absolute-accuracy bound that, per the datasheet, applies when the target fills the field of view; the sub-0.12 °C values reported here are the run-to-run repeatability obtained after a controlled calibration removes the systematic offset, so the two quantities are not in conflict. The calibration is referenced to a Fluke 59 MAX infrared thermometer of nominal accuracy ±2.0 °C or ±2.0% of reading, so the absolute accuracy of the compensated output is bounded by this reference and by the sensor’s ±0.5 °C specification; no claim of absolute accuracy below these limits is made. Accordingly, the sub-0.20 °C RMSE reported here should be read as distance-invariance, the agreement of the compensated output with the reference across the 5–200 cm sweep, and the 0.08–0.12 °C standard deviation as repeatability, rather than as absolute accuracy. Both are robust to the absolute error of the reference: because the same reference is used for calibration and for evaluation, a constant offset at a given setpoint appears in both and cancels in the distance-dependent term. Establishing absolute accuracy traceable to a certified standard would require recalibration against a traceable reference together with a formal uncertainty budget, which we identify as future work. Such an uncertainty analysis should include the uncertainty of the reference thermometer, sensor specification, calibration residuals, repeatability, emissivity effects, distance-to-spot ratio, and environmental conditions.

Table 2 compares the proposed system with six recently reported IR thermometry approaches for precision agriculture [22,49,50,51,52,53], selected to span the platforms in current use rather than to match its point-measurement objective. The systems are contrasted along five criteria (sensor type, distance-compensation method, reported performance metric, operating range, and real-time capability) and differ substantially in purpose. UAV-mounted thermal and RGB platforms [50,53] provide broad spatial coverage but rely on height-specific calibration or temperature-controlled reference panels and on post-flight processing. Ground-based thermal imagers [49,52] and image-segmentation approaches [22] achieve good agreement with reference sensors but entail fixed-geometry or computationally intensive processing less suited to continuous embedded operation. Wireless IR-thermometer networks mounted on center pivots [51] deliver continuous monitoring but depend on existing irrigation infrastructure and do not compensate for stand-off distance. Against this backdrop, the proposed single-pixel system occupies a distinct point in the design space: it integrates ultrasonic distance measurement with lookup-table compensation to maintain a distance-invariant reading across a 5–200 cm range in real time on low-power embedded hardware. The comparison is therefore intended to position the system among alternatives with differing goals, not to rank dissimilar platforms on a single axis.

## 6. Field Measurement on a Live Canopy

**Figure 8:**
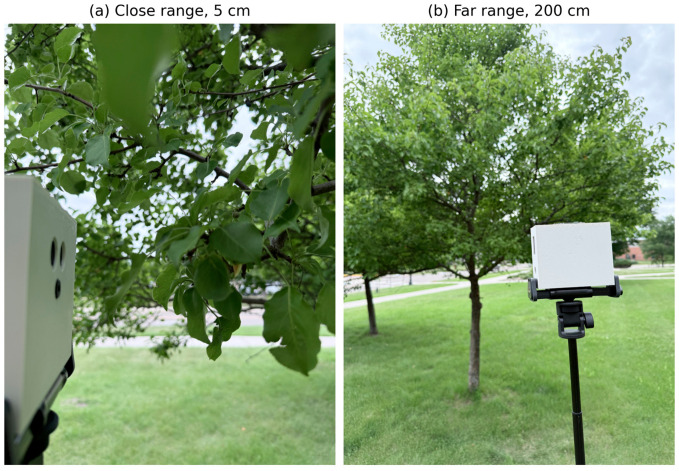
Field measurement setup on a live tree canopy in Brookings, SD: (**a**) close range, 5 cm, with foliage filling the sensor field of view; (**b**) far range, 200 cm.

To assess whether the distance compensation characterized in the laboratory transfers to a vegetated target, a preliminary field measurement was carried out on a live tree canopy on the campus of South Dakota State University (Brookings, SD, USA) under overcast conditions. The instrument was mounted on the same adjustable tripod used on the laboratory bench and aimed at a section of dense foliage (Figure 8). The stand-off distance was stepped through 5, 25, 50, 100, and 200 cm, and the compensated temperature was logged continuously at 1 Hz; the noisy intervals at the start and end of the record correspond to instrument setup and takedown and are excluded from the analysis. The canopy temperature was measured at close range using an external handheld Fluke 59 MAX infrared thermometer, which read 22.2 °C (72 °F).

**Figure 9:**
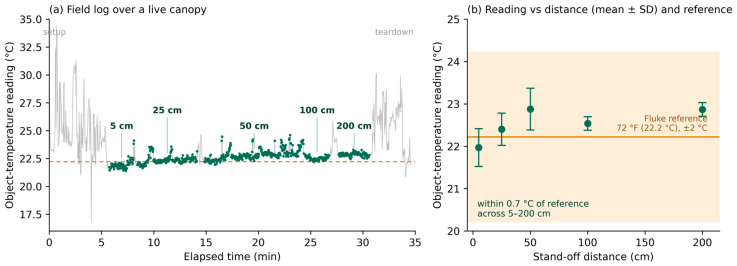
Field measurement on a live canopy: (**a**) the compensated reading logged over the 5–200 cm distance sweep, with the five held distances labeled and the Fluke reference shown as a dashed line; (**b**) the mean reading (±one standard deviation) at each distance, shown against the Fluke reference (solid line) and its ±2.0 °C nominal tolerance (shaded band).

Figure 9 summarizes the field result. Across the full 5–200 cm range, the mean compensated reading varied by only 0.91 °C (from 21.97 °C to 22.88 °C) and agreed with the external handheld reference to within 0.7 °C at every distance, well inside the ±2.0 °C nominal accuracy of the reference. For each held distance, the mean and standard deviation were calculated from the stable portion of the 1 Hz record after excluding setup and transition intervals. No systematic negative drift appeared at the longer stand-off distances, the regime in which an uncompensated single-pixel reading would be expected to fall as the field of view exceeds the foliage and incorporates background radiation; the small residual variation that is present is comparable to the gradual change in canopy temperature over the approximately 25 min acquisition window and does not trend with distance. Within-hold repeatability ranged from 0.16 °C at the longer distances, where the larger footprint averages over more foliage, to 0.45 °C at the closest distance, where the small footprint is more sensitive to leaf-scale temperature structure and motion.

These results provide preliminary evidence that the distance-invariant behavior demonstrated in the laboratory is preserved on this live-canopy target under the tested conditions; they characterize distance compensation and short-term repeatability rather than validated canopy-temperature measurement accuracy. The measurement was acquired on a single overcast day, with a single reference reading at close range and without contact instrumentation of the canopy at each distance; comprehensive field validation across canopy types, leaf densities, fill factors, illumination conditions, wind conditions, environmental temperatures, and water-stress states, with co-located contact references, remains the subject of future work.

## 7. Conclusions

This work presented the design and evaluation of a distance-compensated IR thermometer that integrates a single-pixel IR sensor with ultrasonic ranging and embedded signal processing to correct distance-induced bias. A two-dimensional LUT constructed from regulated setpoints enables a real-time compensation pipeline on a low-power microcontroller. Experimental validation at 15 °C, 30 °C and 60 °C showed that the system reduced distance-dependent errors to residuals bounded within ±0.2–0.3 °C and achieved RMSE values below 0.20 °C across the full 5–200 cm operating range. Repeatability testing across stand-off distances of 20–200 cm confirmed stable, low-noise operation with a precision of 0.08–0.12 °C at 1 Hz sampling.

These results show that distance-dependent bias in single-pixel IR thermometry can be substantially reduced with modest hardware and lightweight firmware, supporting height-flexible operation over the tested 5–200 cm stand-off range. The reported performance reflects distance compensation and short-term repeatability under the tested conditions and should not be read as universally validated canopy-temperature measurement accuracy. The demonstrated distance-invariant RMSE and sub-0.12 °C repeatability were obtained on a uniform high-emissivity reference target under controlled laboratory conditions; a preliminary field measurement on a live tree canopy further provided initial evidence that distance-invariant behavior can be preserved on a vegetated target, with the compensated output agreeing with an external handheld reference to within 0.7 °C across the 5–200 cm range. Future work will address comprehensive outdoor field validation across canopy types, leaf densities, fill factors, illumination conditions, wind conditions, environmental temperatures, and water-stress states; traceable absolute-accuracy validation using a certified temperature reference and formal uncertainty analysis; independent characterization of the LUT interpolation error at non-grid distances; and extension of the validated temperature range.

## Figures and Tables

**Table 1:** Short-term repeatability of the compensated temperature output at 20, 100, and 200 cm for three reference temperature setpoints. Values represent the standard deviation of approximately 300 samples collected at 1 Hz.

Reference Temperature	SD @ 20 cm	SD @ 100 cm	SD @ 200 cm
15 °C	0.08 °C	0.08 °C	0.10 °C
30 °C	0.09 °C	0.10 °C	0.11 °C
60 °C	0.09 °C	0.11 °C	0.12 °C

**Table 2:** Comparison of IR thermometry systems for precision agriculture applications.

Specifications	This Work	[49]	[50]	[51]	[52]	[22]	[53]
Primary use case	Real-time point monitoring at variable stand-off	Time-lapse canopy imaging	Aerial canopy mapping	Pivot-based field monitoring	Greenhouse crop monitoring	Orchard canopy imaging	Calibrated aerial mapping
Sensor Type	Single-pixel IR and ultrasonic	Thermal imager	UAV Thermal and RGB	Wireless IR thermometers	Uncooled TIR camera	Thermal and RGB	UAV Thermal and temp-controlled refs
Distance Compensation	LUT and linear interpolation	None	Segmentation (RGB mask)	None	Real-time ref-panel calibration	Segmentation	Temperature-controlled panels
Reported metric	<0.2 °C (distance bias)	≈1 °C (distance bias)	0.7 °C	≤0.75 °C	±0.62 °C	N/A	1.68 °C (calibrated)
Range	5 to 200 cm	<1.8 m	10–60 m	≈3 m (FOV-based)	Fixed (greenhouse)	Fixed ≈ 0.5 m	40 m
Real-time Operation	Yes	Partial	No	Yes	Yes	Partial/Batch	No
Trade-offs	Single-point; requires specific LUT calibration	No distance compensation	Height specific	Needs pivot infra	Fixed geometry + panels	Complex segmentation	Requires ground setup

## Data Availability

The datasets generated from the current study are available from the corresponding author on reasonable request.
